# Endoxifen in Bipolar Disorder With Hyperammonemia and Renal Impairment: A Case Report

**DOI:** 10.7759/cureus.76839

**Published:** 2025-01-03

**Authors:** Nikhil Gautam, Pallavi Abhilasha

**Affiliations:** 1 Department of Psychiatry, Christian Medical College and Hospital, Ludhiana, IND

**Keywords:** bipolar disorder, encephalopathy, endoxifen, hyperammonemia, mood stabilizer, renal impairment

## Abstract

Lithium, divalproex, and oxcarbazepine are commonly prescribed medications used for the management of bipolar disorder, but each of these drugs comes with its own gamut of adverse effects that make these agents unfavorable in certain medical conditions. Endoxifen, a protein kinase C inhibitor and selective estrogen receptor modulator, originally used in breast cancer treatment, has recently emerged as a potential therapeutic option for managing manic episodes in bipolar disorder. This case report highlights the use of endoxifen in the management of severe manic symptoms of bipolar disorder in a 65-year-old male patient with medical comorbidities arising from traditional mood stabilizer drugs and its potential as an alternative treatment over such drugs.

## Introduction

Bipolar disorder is a relatively common mental health condition. Globally, around 40 million people are living with bipolar disorder, which translates to about 0.53% of the world's population [1]. Management of bipolar disorder at times involves lifelong intake of medications especially in cases with recurrent episodes to prevent relapses. Traditional mood stabilizer drugs such as lithium, divalproex sodium, and oxcarbazepine have been the mainstay of bipolar disorder treatment for the past half a century. Although a lot has been learned in the last 50 years about the pathophysiology and management of bipolar disorder, various guidelines around the world such as the Canadian Network for Mood and Anxiety Treatments (CANMAT), the National Institute for Health and Care Excellence (NICE), and the Indian Psychiatric Society (IPS) still suggest divalproex as the first-line choice in the management of bipolar disorder [2,3]. However, these drugs come with their own gamut of side effects that make them difficult to use in bipolar patients who are medically sick and have multiple comorbidities.

Alternative treatment options include second-generation antipsychotics, such as quetiapine, that have limited efficacy as monotherapy and come with their own subset of side effects [4]. Endoxifen, a protein kinase C inhibitor and selective estrogen receptor modulator, has originally been associated with breast cancer treatment but recently has emerged as a potential therapeutic option for managing manic episodes associated with bipolar disorder. It is given orally in dose formulations of 4 mg and 8 mg, given once daily as monotherapy or in conjugation with a traditional mood stabilizer daily for the treatment of manic episodes in bipolar disorder [5]. The following report highlights a case scenario where the use of the molecule was beneficial for managing bipolar disorder symptoms of the patient when other avenues of treatment were exhausted due to various complications.

## Case presentation

A 65-year-old man, a retired farmer by occupation from a middle-class socioeconomic status, presented to the psychiatry outpatient of a tertiary care institute with complaints of progressively decreased need for sleep, increased irritability, elevated mood with affective lability, overtalkativeness, racing of thoughts, grandiose ideas, and physically abusive behavior in the last one month. A detailed history of the patient revealed that he had been on treatment for bipolar disorder since the age of 29 years and had six manic episodes in the past, experiencing a worsening of his mood symptoms every 3-4 years which were characterized by the presence of elevated mood, aggressive behavior, motor restlessness, grandiose talks, decreased sleep, and increase in appetite. No history suggestive of any substance use in the past was reported.

The patient was maintaining well on medication for two years prior to the current episode. He was taking orally lithium carbonate 600 mg daily for the last 30 years. Additionally, he was on olanzapine 5 mg and clonazepam 1 mg at night time daily from his previous manic episode which occurred two years back. The dose of olanzapine was increased to 10 mg per day at the start of his current manic presentation, and clonazepam 1 mg was replaced with lorazepam 2 mg at night time due to the persistence of sleep disturbance with clonazepam during the manic episode. The patient additionally was a known case of essential hypertension since the year 2012 and was prescribed a combination of amlodipine 5 mg and atenolol 25 mg once daily orally in the morning. 

At the time of admission, the Montgomery-Asberg Depression Rating Scale (MADRS) [6] score was 6 (rating of reduced sleep), and the Young Mania Rating Scale (YMRS) [7] score was 40. MADRS which is comprised of 10 items, each scored from 0 to 6, is used to rate various aspects of depressive symptoms, while YMRS is an 11-item multiple-choice questionnaire with a total score ranging from 0 to 60 that is used to measure the severity of mania in patients of bipolar disorder with a score of more than 20 in YMRS used as inclusion criteria for participation in clinical trials involving manic patients. The scores on both assessments in the present case were suggestive of severe manic episode without any depressive symptoms. Based on the above assessment, a diagnosis of bipolar disorder current episode manic with psychotic symptoms according to the International Classification of Diseases (ICD)-10 [8] was made.

**Figure 1:**
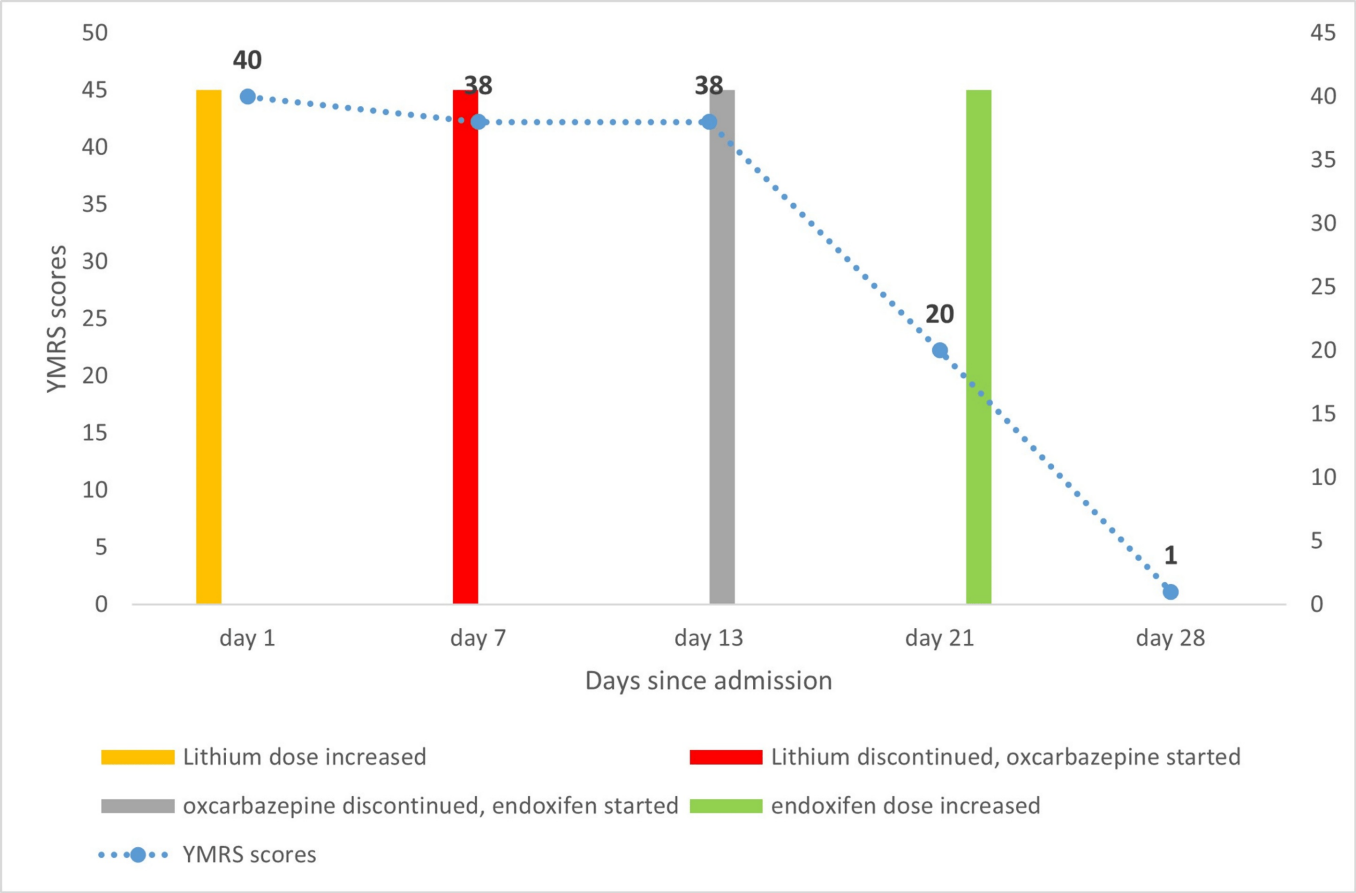
Medication changes and YMRS scores after admission YMRS: Young Mania Rating Scale

The patient was hospitalized in a psychiatric ward for further management. The lithium dose was increased to 900 mg daily on the first day of admission (Figure 1) in view of his worsening manic symptoms and history of good response to lithium carbonate in prior manic episodes. However, after one week of his hospitalization, the patient started developing features of increased agitated behavior, hallucinatory behavior, incomprehensible speech, altered sensorium with impaired cognition, and disorientation to time, place, and person, features synonymous with encephalopathy. Urine output was noted to be decreased.

Laboratory investigation revealed elevated serum creatinine levels of 1.81 mg/dL which at baseline was 1.27 mg/dL on the day of admission (Table 1). The estimated glomerular filtration rate (eGFR) was noted to be 43 mL/min/1.73 m^2^. The serum lithium level was checked seven days after the change was made to increase the dose of lithium and came out to be 1.2 mEq/L. The ammonia level increased to 127.8 mcg/dL which was 80 mcg/dL at admission, a 58% increase from baseline. A urine culture report and ultrasonography of the abdomen suggested findings of benign prostatic hyperplasia with cystitis. In view of worsening sensorium and renal impairment, lithium was switched to oxcarbazepine 600 mg daily dose. The use of sodium valproate was also ruled out as well, as the patient in the past had a history of hyperammonemia on divalproex sodium. Additionally, he was started on oral antibiotics for the management of cystitis.

**Table 1:** Changes in investigation parameters after admission

Days since admission	Serum creatinine (mg/dL)	Serum ammonia (mcg/dL)	Creatinine phosphokinase (U/L)
Day 1	1.27	80	-
Day 7	1.81	127.8	-
Day 13	1.21	480	1078
Day 28	1.21	108	168

Six days after the discontinuation of lithium, serum creatinine levels decreased to 1.21 mg/dL; however, the patient's neuropsychiatric symptoms did not show any sign of improvement. Repeat blood investigations showed increased ammonia levels (480 mcg/dL) and high creatinine phosphokinase levels (1078 U/L). The patient's oxcarbazepine, antipsychotic, and benzodiazepine were stopped. The patient was shifted to a high-dependency unit of the hospital under combined care with the internal medicine department due to his worsening sensorium.

Due to elevated ammonia levels, it was decided to start endoxifen 8 mg in the morning. The patient showed improvement in the above investigative parameters over one week after the change in management, but his disturbed circadian rhythm, elevated psychomotor activity, irritable mood, and agitated behavior continued. However, he was discharged from the hospital on his attendant's request due to financial constraints but was advised to continue with endoxifen at home which was further increased to 16 mg per day at the time of discharge. During the follow-up visit after one week of discharge, the patient showed marked improvement in his mood symptoms. YMRS scores had reduced significantly to a value of 1 showcasing remission of manic symptoms in the patient. No adverse effects were reported during subsequent visits by the end of two months, and the patient continued to stay in remission on endoxifen as the sole drug for managing his bipolar disorder till his last visit.

## Discussion

Bipolar disorder can be a severely disabling psychiatric disorder with a lifetime prevalence of 1% [9]. Of the available drugs that are currently approved for the treatment of bipolar disorder, all are associated with adverse effects and issues in tolerability that make them unfavorable in certain conditions. Lithium carbonate has been associated with renal side effects such as increased micturition along with diabetes insipidus, deranged creatinine, and acute renal failure [10,11], while divalproex and oxcarbazepine are associated with hepatic impairment and hyperammonemia [12,13].

The clinician at times might find themselves in a dilemmatic situation where they are forced to use alternative agents due to adverse effects or lack of efficacy. Advances in molecular biology have shown that bipolar disorder is in fact an issue of overactive intracellular signaling involving protein kinase C [13]. The aforementioned drugs such as lithium and valproate are also associated with protein kinase C inhibition. Endoxifen, an active metabolite derived from tamoxifen as mentioned previously, is also a potent protein kinase C inhibitor. The efficacy and safety of endoxifen are shown in recent multicenter clinical trials for the management of bipolar disorder done by Ahmad et al. which concluded that 8 mg of endoxifen was efficacious in acute manic symptoms in patients with bipolar disorder. Additionally, an early time to remission of the disease was observed with endoxifen compared to divalproex along with no drug-associated withdrawals [14]. Furthermore, it does not have the pitfalls associated with other drugs used for treating bipolar disorder like divalproex and lithium discussed earlier, thus showing potential in patients who have impaired renal and liver functions. 

Our case report highlights one such niche scenario where other agents were contraindicated and the use of endoxifen showed a positive outcome. Furthermore, regular monitoring of liver and renal parameters did not show any worsening; on the contrary, these parameters improved significantly by the time the patient was discharged. No relapse of symptoms was also observed by the end of two months.

## Conclusions

This case showcases the role of endoxifen (8-16 mg per day) as a ray of hope in the management of manic episodes in bipolar patients with renal and hepatic impairment where other drugs are contraindicated. Patients who would otherwise be left with limited options can greatly benefit from its promising usage. Future studies should explore the utility of endoxifen in bipolar patients with medical comorbidities and endoxifen's positioning in future treatment guidelines for bipolar disorders.
